# Complement Factor D (adipsin) Levels Are Elevated in Acquired Partial Lipodystrophy (Barraquer–Simons syndrome)

**DOI:** 10.3390/ijms22126608

**Published:** 2021-06-21

**Authors:** Fernando Corvillo, Laura González-Sánchez, Alberto López-Lera, Emilia Arjona, Giovanni Ceccarini, Ferruccio Santini, David Araújo-Vilar, Rebecca J Brown, Joan Villarroya, Francesc Villarroya, Santiago Rodríguez de Córdoba, Teresa Caballero, Pilar Nozal, Margarita López-Trascasa

**Affiliations:** 1 Complement Research Group, Hospital La Paz Institute for Health Research (IdiPAZ), La Paz University Hospital, 28046 Madrid, Spain; laura.gonzalez@ciberer.es (L.G.-S.); alberto.lopez@ciberer.es (A.L.-L.); pilar.nozal@salud.madrid.org (P.N.); margarita.lopez@uam.es (M.L.-T.); 2 Center for Biomedical Network Research on Rare Diseases, 28029 Madrid, Spain; earjona@cib.csic.es (E.A.); srdecordoba@cib.csic.es (S.R.d.C.); mteresa.caballero@ciberer.es (T.C.); 3 Department of Molecular Biomedicine, Margarita Salas Center for Biological Research, 28040 Madrid, Spain; 4 Obesity and Lipodystrophy Center at the Endocrinology Unit, Department of Clinical and Experimental Medicine, University Hospital of Pisa, 56126 Pisa, Italy; giovanni.ceccarini@unipi.it (G.C.); ferruccio.santini@unipi.it (F.S.); 5 UETeM-Molecular Pathology Group, Department of Psychiatry, Radiology, Public Health, Nursing and Medicine, IDIS-CIMUS, University of Santiago de Compostela, 15703 Santiago de Compostela, Spain; david.araujo@usc.es; 6 National Institute of Diabetes and Digestive and Kidney Diseases, National Institutes of Health, Bethesda, MD 20814, USA; brownrebecca@niddk.nih.gov; 7 Departament de Bioquimica I Biomedicina Molecular, Institut de Biomedicina de la Universitat de Barcelona, 08007 Barcelona, Catalonia, Spain; jvillarroya@ub.edu (J.V.); fvillarroya@ub.edu (F.V.); 8 CIBER Fisiopatología de La Obesidad Y Nutrición, 28029 Madrid, Spain; 9 Department of Allergy, La Paz University Hospital, 28046 Madrid, Spain; 10 Hospital La Paz Institute for Health Research (IdiPAZ), 28046 Madrid, Spain; 11 Immunology Unit, La Paz University Hospital, 28046 Madrid, Spain; 12 Department of Medicine, Universidad Autónoma de Madrid, 28049 Madrid, Spain

**Keywords:** adipsin, complement factor D, acquired partial lipodystrophy, Barraquer–Simons syndrome, complement system

## Abstract

Complement overactivation has been reported in most patients with Barraquer–Simons syndrome (BSS), a rare form of acquired partial lipodystrophy. Complement Factor D (FD) is a serine protease with a crucial role in the activation of the alternative pathway of the complement system, which is mainly synthesized by adipose tissue. However, its role in the pathogenesis of BSS has not been addressed. In this study, plasma FD concentration was measured in 13 patients with BSS, 20 patients with acquired generalized lipodystrophy, 22 patients with C3 glomerulopathy (C3G), and 50 healthy controls. Gene expression and immunohistochemistry studies were assayed using atrophied adipose tissue from a patient with BSS. We found significantly elevated FD levels in BSS cases compared with the remaining cohorts (*p* < 0.001). There were no significant differences in FD levels between sexes but FD was strongly and directly associated with age in BSS (*r* = 0.7593, *p =* 0.0036). A positive correlation between FD and C3 was seen in patients with C3G, characterized by decreased FD levels due to chronic C3 consumption, but no correlation was detected for BSS. Following mRNA quantification in the patient’s adipose tissue, we observed decreased ***C****FD* and *C3* but elevated *C5* transcript levels. In contrast, the increased FD staining detected in the atrophied areas reflects the effects of persistent tissue damage on the adipose tissue, thus providing information on the ongoing pathogenic process. Our results suggest that FD could be a reliable diagnostic biomarker involved in the pathophysiology of BSS by promoting unrestrained local complement system activation in the adipose tissue environment.

## 1. Introduction

Lipodystrophies are a heterogeneous group of rare disorders characterized by the loss of adipose tissue [1]. There are multiple subtypes of lipodystrophies, which can be congenital or acquired and which in turn vary in the distribution of adipose tissue loss, being partial, generalized, or localized. One of these diseases is Barraquer–Simons syndrome (BSS; ORPHA:79087), an acquired form of partial lipodystrophy characterized by bilateral symmetrical loss of adipose tissue at upper body locations [1]. Dysregulation of the alternative pathway (AP) of the complement system, denoted by C3 hypocomplementemia, is a frequent clinical feature among patients with BSS, commonly due to the presence of C3 nephritic factor (C3NeF) [2]. C3NeF is an autoantibody directed to the AP C3 convertase that acts by stabilizing and increasing the half-life of the complex, which leads to C3 consumption and chronic C3 hypocomplementemia [3]. In most of the published cohorts of patients with BSS, C3NeF is the most frequent antibody (70%) [2,4], although our group has described the existence of additional autoantibodies against AP proteins [2]. Moreover, Mathieson et al. evidenced that C3NeF could lyse adipocytes [5].

C3NeF is not exclusive to patients with BSS, but is also involved in another pathological condition not related to lipodystrophy called C3 glomerulopathy (C3G) [6]. The mechanistic connection with complement activation is further reinforced by the fact that 20% of BSS patients eventually develop C3G [1]. Our group has recently published the first evidence of ongoing complement system activation in affected adipose tissue from an 11-year-old girl with BSS [7]. This finding, together with the abnormal levels of the AP proteins in patients with BSS, points to complement dysregulation as a central pathological event in the development of this rare disease; however, the exact mechanism by which complement causes fat loss remains unclear.

Complement factor D (FD), also termed adipsin, is a serine protease with a crucial role in the activation of the AP of the complement system [8,9]. FD is secreted by several tissues and cell types, although the major source in humans is mature adipocytes and macrophages in adipose tissue [8,9,10]. Given the connection of FD with adipose tissue and complement system activation, it could be considered a central player in the pathogenesis of BSS, as previously suggested by other authors [4,5,11]. However, this assumption has not been demonstrated yet. In pathological situations where adipose tissue function is widely affected, such as lipodystrophy syndromes, the synthesis and secretion of the adipokines leptin and adiponectin are seriously impaired [1], but this is unknown for FD. Recently, Wu and collaborators showed that FD levels were reduced but still detected in patients with congenital and acquired generalized lipodystrophy (CGL and AGL, respectively), in contrast to patients with familial partial lipodystrophy (FPLD), in whom FD levels were similar to healthy subjects [12]. However, the authors did not quantify FD levels in patients with BSS nor did they analyze the existence of local differences in its expression levels and, therefore, the role of FD in the pathogenesis of the BSS remains unanswered.

The present study aimed to quantify FD levels in a cohort of 13 patients with BSS and compare them with patients in other pathological situations, such as AGL and C3G, and with healthy donors. Another objective of this study was to explore the effect of age and sex on FD levels in the previously mentioned cohorts. Finally, gene expression studies and detection of FD were carried out on adipose tissue from a patient with BSS previously studied by our group.

## 2. Results

### 2.1. Circulating FD Levels Are Increased in Patients with BSS

**Figure 1:**
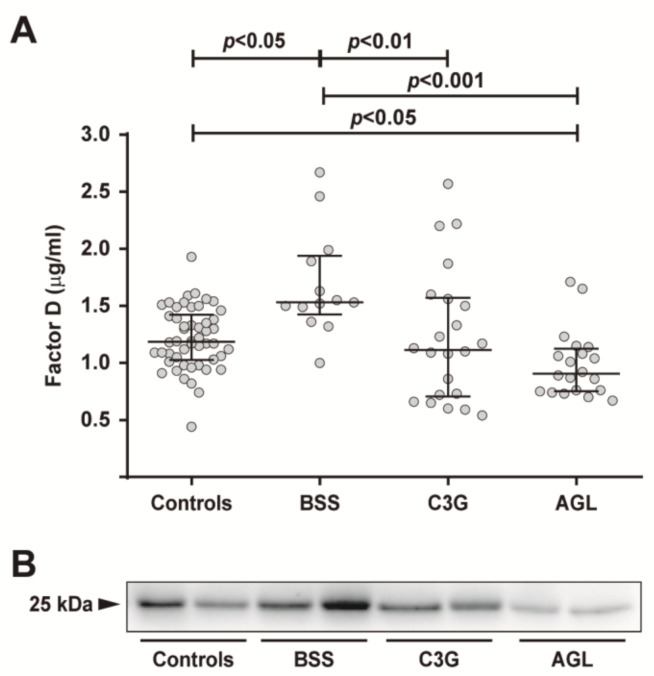
Plasma FD levels. (**A**) FD levels were quantified in plasma samples from controls (*n* = 50), patients with Barraquer–Simons syndrome (BSS, *n* = 13), patients with C3 glomerulopathy (C3G, *n* = 22), and patients with acquired generalized lipodystrophy (AGL, *n* = 20). The results are presented as medians and interquartile ranges. Differences between groups were assessed by the Kruskall–Wallis test. *p <* 0.05 was considered statistically significant. (**B**) FD levels were assessed by Western blot, confirming that FD was elevated in patients with BSS and decreased in patients with AGL.

The plasma FD concentration was measured in 13 patients with BSS, 20 patients with AGL, 22 patients with C3G and 50 healthy donors. FD levels were significantly elevated in patients with BSS compared with individuals from the control, AGL and C3G cohorts (BSS: median 1.530 µg/mL (IQR—1.425–1.940); controls: 1.185 µg/mL (1.025–1.423); AGL: 0.905 µg/mL (0.753–1.125); C3G: 1.115 µg/mL (0.705–1.570); *p <* 0.05 for controls and C3G, and *p* < 0.001 for AGL) (Figure 1). In contrast, plasma FD levels in the AGL cohort were significantly lower than those of controls (*p <* 0.05) (Figure 1). We performed a Western blot on the plasma from controls, patients with BSS, C3G and AGL and observed that FD levels were reduced in patients with AGL and elevated in BSS compared to controls. Demographic details and basic clinical information are listed in Table 1.

### 2.2. FD Levels Are a Potentially Reliable Biomarker for the Diagnosis of BSS

**Figure 2:**
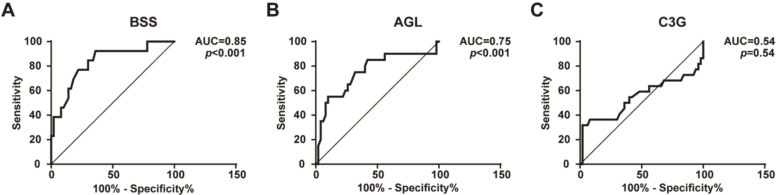
Plasma FD levels ROC curves for samples from patients with Barraquer–Simons syndrome (BSS) (**A**), patients with acquired generalized lipodystrophy (AGL) (**B**), and patients with C3 glomerulopathy (C3G) (**C**). *p <* 0.05 was considered statistically significant. AUC, area under the ROC curve.

A ROC curve using FD levels was implemented to find a cut-off value for predicting the utility of this parameter as a diagnostic biomarker. ROC curve analysis indicated that plasma FD levels were a potential diagnostic biomarker for BSS and AGL, with Area Under the Curve (AUC) values of 0.83 (95% confidence interval (CI): 0.70–0.95, *p* < 0.001) (Figure 2A) and 0.75 (0.61–0.89, *p <* 0.001) (Figure 2B), respectively. In contrast, in patients with C3G, FD levels were not a reliable biomarker, with an AUC value of 0.54 (0.37–0.71, *p =* 0.54) (Figure 2C). The cut-off was chosen using the Youden index. An FD level value ≥1.47 µg/mL was associated with a higher probability to detect patients with BSS (sensitivity: 77%, specificity: 78%, negative predictive value: 77%, positive predictive value: 78%, likelihood ratio: 3.47). However, the validation of the cut-off value ≤0.92 µg/mL for patients with AGL did not show acceptable results (sensitivity: 15%, specificity: 33%, negative predictive value: 45%, positive predictive value: 10%, likelihood ratio: 0.23).

### 2.3. Age- and Sex-Related Differences in FD Levels

**Figure 3:**
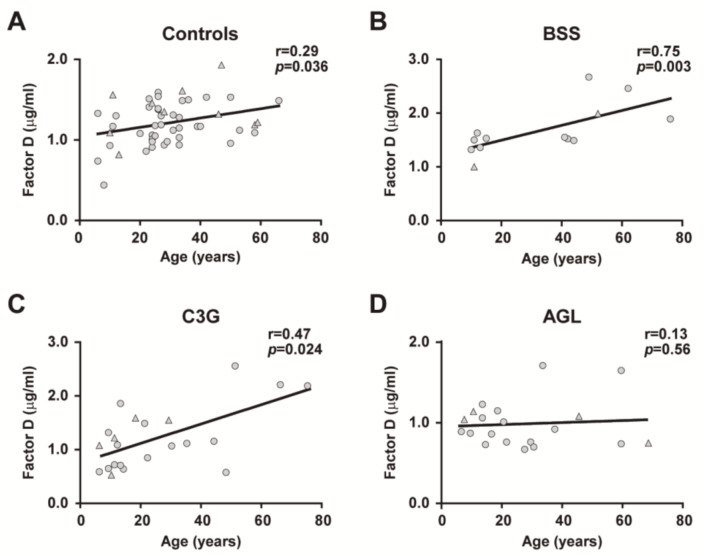
Correlation of plasma FD levels with age. Age positively correlated with FD levels from controls (**A**), patients with Barraquer–Simons syndrome (BSS) (**B**), patients with C3 glomerulopathy (C3G) (**C**), but not in patients with acquired generalized lipodystrophy (AGL) (**D**). These correlations were calculated using the Spearman Rank correlation coefficient. Females are indicated by circles and males by triangles. *p <* 0.05 was considered to be statistically significant.

**Figure 4:**
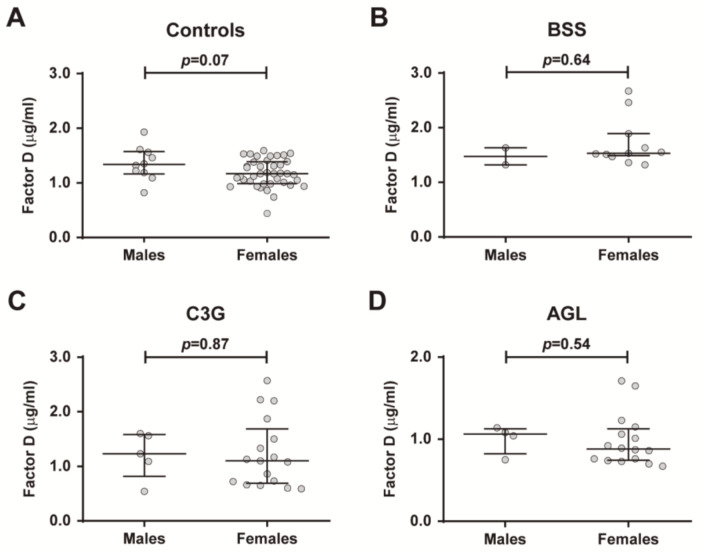
Differences in plasma FD levels between sexes. FD levels were compared between males and females in controls (**A**), patients with Barraquer–Simons syndrome (BSS) (**B**), patients with C3 glomerulopathy (C3G) (**C**), and patients with acquired generalized lipodystrophy (AGL) (**D**). These comparisons were assessed using the Mann–Whitney test. The results are represented as medians and interquartile ranges. *p <* 0.05 was considered to be statistically significant.

We analyzed the effect of age on the concentration of FD in all cohorts. Linear regression analyses demonstrated a significant age-related effect for FD levels in controls, BSS and C3G cohorts, but not for patients with AGL. Age was significantly and positively correlated with FD levels in healthy controls (*r =* 0.2962, *p =* 0.0368, Figure 3A), patients with BSS (*r =* 0.7593, *p =* 0.0036, Figure 3B) and patients with C3G (*r =* 0.4782, *p =* 0.0244, Figure 3C), but not in patients with AGL (Figure 3D). No significant differences were observed in FD levels between males and females in all cohorts (Figure 4A–D).

### 2.4. Effects of Complement System Activation on Plasma FD Levels

**Figure 5:**
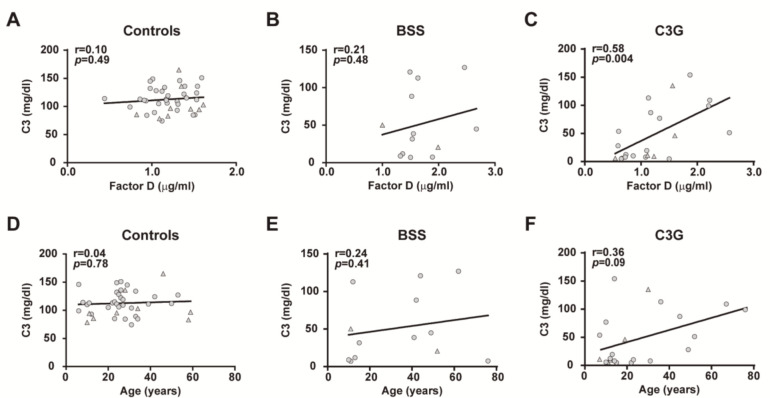
Correlation between C3 and FD levels in plasma and between C3 levels and age. The relationship between these parameters was investigated in controls (**A**,**D**), patients with Barraquer–Simons syndrome (BSS) (**B**,**E**) and patients with C3 glomerulopathy (C3G) (**C**,**F**). Correlations were analyzed using the Spearman Rank correlation coefficient. Females are indicated by circles and males by triangles. *p <* 0.05 was considered to be statistically significant.

**Figure 6:**
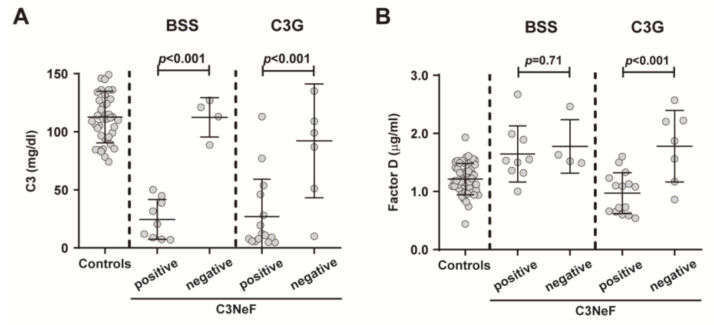
Differences in C3 and FD plasma levels in patients with Barraquer–Simons syndrome (BSS) and patients with C3 glomerulopathy (C3G). C3 (**A**) and FD (**B**) levels were analyzed in the BSS and C3G cohorts according to the presence of C3 nephritic factor (C3NeF). The results are represented as medians and interquartile ranges. *p <* 0.05 was considered to be statistically significant.

To better understand the differences seen in FD levels between patients with BSS and patients with C3G, we assessed the correlation between circulating C3 and FD levels. Our results showed that FD correlated significantly with C3 in patients with C3G (*r =* 0.5863, *p =* 0.0041), while no correlation was observed in patients with BSS and controls (Figure 5A–C). In addition, we studied the relationship between C3 levels and age, and no correlation was observed in any cohort (Figure 5D–F). We then compared FD levels between patients with positive/negative results for C3NeF autoantibodies in both the BSS and C3G cohorts. Patients with a positive result for C3NeF had significantly lower serum C3 levels in both cohorts (*p <* 0.001) (Figure 6A), although differences in FD concentration were found only in patients with C3G, being lower in those with C3NeF autoantibodies (C3NeF positive: 1.080 µg/mL (0.650–1.230), and C3NeF negative: 1.870 µg/mL (1.170–2.220); *p <* 0.001; Figure 6B).

### 2.5. Increased FD Labelling at Lipoatrophic Areas

**Figure 7:**
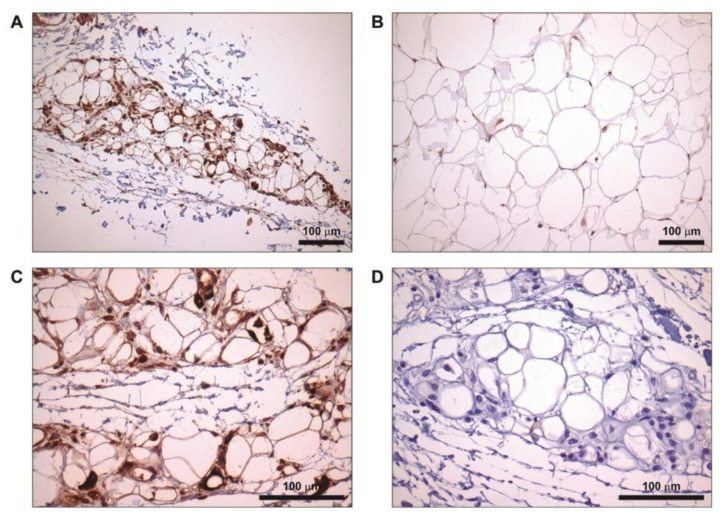
Immunostaining of FD in adipose tissue. The subcutaneous adipose tissue of a patient with Barraquer–Simons syndrome (**A**) showed higher and widely spread staining of FD in comparison with healthy control tissue (**B**) (immunohistochemistry, original magnification ×20). More detailed images from atrophied areas showed that FD staining was detected both in the stroma vascular fraction and in adipocytes (**C**) (immunohistochemistry, original magnification ×40). Staining was performed using a rabbit IgG (ab172730; Abcam) as an isotype control (**D**) (immunohistochemistry, original magnification ×40). Scale bars: 100 µm.

We performed an immunohistochemistry assay to analyze the presence and localization of FD in the adipose tissue from an 11-year-old girl with BSS and two healthy donors (a 69-year-old male and a 57-year-old female). We observed that FD was overrepresented and widely extended along affected areas of adipose tissue from the patient with BSS (Figure 7A) compared with adipose tissue from a healthy donor, in which FD was observed in small isolated spots (Figure 7B). Using a higher magnification image, we detected that, although most FD staining was present in the extracellular space and the stroma vascular cells, there were at least some adipocytes that show remarkable cytoplasmic staining (Figure 7C). To determine whether the results were due to nonspecific binding of the primary antibody, we performed isotype control staining (Figure 7D). This result prompted us to perform gene expression analyses in the same adipose tissue biopsies. Levels of transcripts corresponding to components of the complement system *C3* and *Complement Factor D* (*CFD*) showed reduced expression in adipose tissue from the patient with respect to healthy controls; in contrast, *C5* mRNA was marginally increased (Table 2). Furthermore, the examination of gene expression levels of master regulatory factors associated with the promotion of adipogenesis indicated that peroxisome proliferator-activated-γ (*PPARG*) and CCAAT/enhancer-binding protein α (*CEBPA*) mRNAs were down-regulated in the patient with BSS with respect to controls (Table 2).

## 3. Discussion

In this study, we found that plasma FD levels are significantly higher in a representative cohort of patients with BSS compared with controls and another two cohorts composed of patients with AGL and C3G. This study points to FD as a potential biomarker in BSS.

At the end of the 1980s and the beginning of the 1990s, Dr. Spiegelman’s group showed that mouse adipocytes secreted a tissue-specific adipokine that they called adipsin, which has important functions for the development of adipose tissue [9]. This protein was found to be identical to human FD, which is considered to be a rate-limiting factor in the activation of the AP of the complement system [9]. In this pathway, FD cleaves complement factor B and catalyzes the formation of C3 convertase (C3bBb) [13], which leads to a proteolytic cascade that produces several complement fragments, including C3a, C3b, C5a, and C5b, and eventually leads to the formation of the membrane attack complex (C5b-9) [13].

The role of the complement system in the biology of adipose tissue has been described in several papers [14,15,16]. The fact that FD is the only protein of the complement system that is mainly synthesized by adipocytes, in contrast to the other complement components, which are produced by the liver, places FD as an important player in adipose tissue function. Adipocytes are also capable of synthesizing other complement components, particularly those involved in the initial steps of complement activation (e.g., factors B, C2, C3, C4, C1Q, C1R, C1S) but they express almost none of the terminal complex components (C8A, C8B, C8G, C9), with the exception of C5, C6, and C7 [17,18,19,20]. Furthermore, local complement activation promotes lipid accumulation and adipocyte differentiation [21].

Increased levels of serum FD are closely related to several pathological conditions. For example, plasma FD has been found to be elevated in patients with obesity [22,23,24], age-macular degeneration [25], coronary artery disease [26], osteoarthritis [27], non-alcoholic fatty liver disease [28], and polycystic ovary syndrome [29]. Conversely, according to different experimental and clinical studies, plasma FD concentrations are low in animals and patients with diabetes mellitus [30,31,32,33], and in those pathological conditions where adipose tissue is almost completely absent, such as CGL and AGL [12], in agreement with the results from our group (Figure 1). On the other hand, plasma FD concentrations were reported to be normal in patients with FPLD, in whom some adipose tissue is preserved [12]. Herein, we provide experimental evidence for altered FD plasma levels in patients with BSS and C3G, two conditions characterized by dysregulation of the AP of the complement system. Interestingly, while BSS patients exhibited significantly elevated FD concentrations, those with C3G showed a slight decrease in plasma FD (Figure 1). Additionally, our results suggest that FD levels may be a marker to identify patients with BSS (Figure 2A). Specifically, FD levels ≥1.47 µg/mL were associated with a higher probability of detecting patients with BSS. In contrast, FD levels were not a useful biomarker in patients with AGL (Figure 2B), which is consistent with the low FD levels typically found in all generalized lipodystrophies.

Moreover, we observed a significant direct correlation of FD levels with age in healthy controls, BSS patients and C3G patients, with this correlation being stronger in the BSS cohort (Figure 3A–C). In striking contrast to this observation, the complete absence of adipose tissue that characterizes AGL could help explain the lack of age-dependence of FD levels in patients with this generalized form of lipodystrophy (Figure 3D). Additionally, no noticeable effect of sex on FD levels in the control, BSS and C3G cohorts was observed (Figure 4A–D).

FD is constitutively secreted at high rates by adipose tissue but rapidly catabolized in the proximal renal tubules [9,34]. The fractional catabolic rate of plasma FD has been estimated to be 60% per hour, contributing to its low serum concentrations in non-pathological situations [35]. Serum FD levels were found to be elevated in patients with chronic renal failure due to a lower renal catabolic rate [35,36,37]. In this work, we did not observe differences in FD concentrations between patients with C3G and controls (Figure 1); however, a positive correlation between FD and C3 was seen in patients with C3G, a condition characterized by decreased FD levels due to chronic C3 consumption (Figure 5). Surprisingly, no such FD/C3 correlation was detected in BSS, a clinical condition also characterized by complement hyperactivation (Figure 5). When we analyzed FD in situations of complement dysregulation, such as in the presence of C3NeF, plasma FD was significantly lower in C3NeF-positive patients with C3G compared with the rest of the negative individuals (Figure 6B,C). In contrast, complement system activation did not affect FD levels in patients with BSS (Figure 6B,C). In a previous work, Gaya da Costa et al. showed that FD levels were not related to either the activity of the AP or C3 levels in a healthy population [38], in agreement with our results (Figure 5). In contrast to other AP proteins, FD does not undergo proteolytic activation to justify its consumption during AP activation. There are studies about FD levels in systemic lupus erythematosus (SLE), another condition characterized by pathological activation of the complement system, in which plasma levels of FD were not different from those of healthy controls [39,40]. Complement activation in SLE is mainly due to the classical pathway, with limited AP implication, is and normally not as pronounced as in C3G, which may explain why patients with SLE do not show a decrease in plasma FD levels. FD is almost completely reabsorbed in the renal tubule, but in situations where a selective defect of the tubular epithelium occurs, such as Fanconi’s syndrome, FD levels were elevated in urine and decreased in plasma [36]. C3G is a chronic disease that tends to progress to end-stage renal disease and tubular atrophy in advanced cases [41]. C3NeF induces a marked activation of the AP, which usually appears even before renal manifestations. This chronic activation of the complement system causes massive production of complement fragments which are deposited in the kidney, severely damaging the organ and leading to tubular dysfunction and renal failure [41]. Defective tubular reabsorption could therefore explain the lower levels of FD found in C3NeF-positive patients with C3G. However, further studies are needed to understand whether this is the case or whether the lower FD levels are mediated by different mechanisms secondary to complement activation.

In our BSS cohort, three out of thirteen patients (23%) had kidney disease (Table 1), and all of them showed elevated levels of plasma FD in contrast to those observed in patients with C3G. Moreover, as we have previously clarified, in patients with BSS, FD levels do not correlate with the degree of activation of the complement system. Therefore, one possible explanation of our results is that adipose tissue of patients with BSS expresses more FD than unaffected adipose tissue, resulting in significantly elevated circulating levels. To further examine this hypothesis, we studied the presence of FD in the adipose tissue from the affected arm of an 11-year-old girl with BSS, observing that FD in the atrophied areas was significantly overrepresented compared to healthy donor tissue (Figure 7A,B). Although most FD staining was present in the extracellular space and the stroma vascular cells, some adipocytes showed remarkable cytoplasmic staining (Figure 7A,C). Considering these results, the stroma vascular fraction could have a determining role in the synthesis of complement components in the pathogenic context of BSS, but further experiments will be necessary to confirm this hypothesis. In a previous report from our group, we demonstrated ongoing complement activation by detecting local C3, C5a, and C5b-9 deposits in adipose tissue from this same patient [7]. In this context, if FD production is higher than usual, further activation of the AP could occur locally in the adipose tissue, and combined with the presence of C3NeF, damage of the tissue would occur. However, following mRNA quantification in the patient’s adipose tissue, we observed decreased *CFD* and *C3* but elevated *C5* transcript levels as compared to those from the adipose tissue of a control female obtained from the same anatomical location (Table 2). Although these results are somewhat conflicting with respect to those obtained by immunohistochemistry, they could be explained by the lower expression of *PPARG* and *CEBPA* regulatory genes observed in the patients’ samples, a scenario consistent with lipoatrophy (Table 2) [42]. In agreement with this, the down-regulation of *PPARG* may be the main causative event of the coordinate impairment in the expression of *CFD* and *C3* genes, which are known targets of *PPARG*-dependent regulation [43,44]. Correspondingly, if the tissue sample was obtained at an advanced stage of lipodystrophy, the expression of complement genes may be significantly influenced by down-regulation of *PPARG* and *CEBPA*. However, the elevated expression of C5 (Table 2), a gene not controlled by *PPARG*, may suggest that upregulation of complement-related genes is a chronic phenomenon occurring before and during adipose tissue damage. Nevertheless, we cannot rule out that the differences we observed between the controls and the patient may be due to a lack of age matching. Previous data showed that C5, but not C3 levels, increase with age [38], and as we demonstrated in this work, FD concentration increased with age in controls. According to this higher *CFD* and *C5* mRNA levels would be expected in controls as compared to the patient. Our finding of reduced expression of the *CFD* gene in the patient is in striking contrast to bibliography-based expectations, but conversely, we observed higher *C5* expression in the patient´s tissue, which could partially support our hypothesis. However, we cannot rule out that the lower *CFD* expression in the BSS is a direct consequence of the down-regulation of the *PPARG* gene, or that it is due to the lack of appropriately age-matched controls. On the other hand, our immunohistochemistry results may instead reflect (i) a lack of correlation between FD and C3 mRNA/protein levels in this pathological context and (ii) the effects of persistent tissue damage on the adipose tissue, thus providing information on the ongoing pathogenic process. Moreover, in light of these observations, we cannot rule out the contribution of other non-adipose cell types to the adipocyte milieu of complement proteins. For these hypotheses to be tested, fat biopsies from affected and unaffected areas should be obtained during the progression of the disease, which is a major and evident limitation.

In conclusion, the findings herein presented suggest that FD could be a reliable diagnostic biomarker involved in the pathophysiology of BSS by promoting unrestrained local complement system activation in the adipose tissue environment. Further studies are needed to validate its importance in the mechanism of the disease, as well as a possible treatment target.

## 4. Materials and Methods

### 4.1. Study Cohorts

Thirteen patients with BSS and 20 patients with AGL were enrolled for this study. All of them were diagnosed based on standardized criteria [45]. Briefly, one criterion for both lipodystrophies was fat loss during childhood or adulthood. The patients with BSS were characterized by large areas of the body being affected, particularly the head, shoulders, upper extremities, and trunk, with preservation of adipose tissue in the lower extremities. In contrast, patients with AGL were characterized by loss of fat, affecting the entire body. The presence of autoimmune markers or autoimmune diseases can be supportive of the diagnosis of BSS and AGL. Laboratory findings such as low complement C3 and the presence of C3NeF were used during the diagnosis of BSS.

Congenital forms of lipodystrophy were excluded based on the natural history of the disease, clinical features, age at onset, and/or pathogenic variants in CGL (*AGPAT2, BSCL2, CAV1, PTRF, PPARG*) and FPLD-related genes (*LMNA, PPARG, PLIN1, CIDEC, LIPE, CAV1, AKT2*). No consanguinity was reported in any case. The clinical, demographical, and immunological features for these 13 patients with BSS were previously described in [2].

Twenty-two patients with biopsy-proven C3G were selected for inclusion in this study based on standardized criteria [6,46,47]. Additionally, 50 healthy subjects were recruited as controls.

All cohorts were matched in terms of sex and age. For more details, see Table 1.

### 4.2. Biological Samples

Blood samples were drawn in plain tubes, allowed to clot at room temperature, and centrifuged for 10 min at 4 °C. EDTA-plasma was aliquoted and stored at –20 and –80 °C until use to avoid repeated freezing and thawing.

Immunohistochemistry and gene expression studies were performed using subcutaneous adipose tissue biopsy from the arm of an 11-year-old girl with BSS (described in [7]). As controls, we collected subcutaneous adipose tissue from two healthy, lean, male and female (69- and 57-year-olds, respectively) obtained at similar anatomical sites (arm). Adipose samples from these healthy controls has been described elsewhere [48]. The tissues used for histological studies were stored in paraffin blocks. Fresh frozen tissues were preserved until their use to extract mRNA.

### 4.3. Complement System Determinations

Serum C3 levels were measured by nephelometry (Siemens Healthcare, Erlangen, Germany). C3NeF was detected by ELISA assay as previously described [49].

### 4.4. Measurement of FD in Plasma by ELISA

Plasma FD was measured using an in-house ELISA assay, optimized for use in plasma. ELISA plates (MaxiSorp^®^, Nunc) were coated with 100 ng/well of mouse monoclonal anti-human FD (GAU 01-04-02, Invitrogen, Carlsbad, CA, USA) diluted in carbonate-bicarbonate buffer pH 9.3 (overnight, 4 °C). Plates were blocked with PBS-BSA 1% and washed with PBS-BSA 0.1% (both incubations 1 h at 37 °C). A standard curve ranging from 0 to 80 ng/mL of purified FD (Complement Technology, Tyler, TX, USA) was included on each plate, alongside one internal control plasma sample used to assess interassay variability. The interassay coefficient of variation was below 10%. Plasma samples were diluted in PBS-BSA 0.1% at 1/50, 1/100, and 1/200 dilutions for analysis. FD detection was made using a mouse monoclonal anti-human FD conjugated with biotin (GAU 008-01B-02, Invitrogen) (1/2500 dilution, 1 h at 37 °C) and then with streptavidin-peroxidase polymer (S2438, Sigma Aldrich, St Louis, MO, USA) (1/400, both incubations 45 min at 37 °C). The reactions were developed using ABTS as substrate and read at 405/620 nm using an Epoch™ Microplate Spectrophotometer (BioTek Instruments, Inc., Winooski, VT, USA). All samples were measured in duplicate.

### 4.5. Western Blot Analysis

Plasma underwent electrophoresis in SDS-PAGE gels and was transferred to nitrocellulose membranes using iBlot 2 Dry Blotting System (Invitrogen). They were incubated with anti-FD antibody (PA5-79034, Invitrogen) (1/1000 dilution, overnight at 4 °C) and then with goat anti-rabbit IgG (#107-6515, Bio-Rad, Hercules, CA, USA) (1/40,000 dilution, 1 h at room temperature). Peroxidase activity was analyzed using ECL™ Select Western Blotting Detection Reagent (GE Healthcare Bio-Sciences, New Burnswick, NJ, USA). UVITEC Alliance 1D MAX (UVITEC Cambridge) was used for image acquisition.

### 4.6. RNA Extraction and qRT-PCR

Adipose tissue samples were fragmented by mechanical disruption and then homogenized in RA-1 buffer (Macherey-Nagel, Düren, Germany) supplemented with 10% β-mercaptoethanol. RNA was immediately isolated from the homogenate using a column affinity-based methodology (NucleoSpin^®^ RNA II; Macherey-Nagel). cDNA was synthesized from 0.5 µg of total RNA using MultiScribe reverse transcriptase and random hexamer primers (Applied Biosystems, Foster City, CA, USA). mRNA expression levels were determined by quantitative real-time reverse transcription-polymerase chain reaction (qRT-PCR) using TaqMan reagents and an Applied Biosystems 7500 Fast Real-Time PCR System (Applied Biosystems). Real-time PCR was performed in a final volume of 20 µL using TaqMan Universal PCR Master Mix, No AmpErase UNG reagent, and the following specific primer probes (TaqMan Gene Expression Assays; Applied Biosystems): *C3* (Complement component 3), Hs00163811_m1; *C5* (Complement component 5), Hs01004342_m1; *CFD* (Complement factor D, adipsin), Hs00157263_m1; *PPARG* (peroxisome proliferator-activated receptor-gamma, *PPAR-γ*), Hs00234592_m1; *CEBPA* (CCAAT enhancer-binding protein-alpha), Hs00269972_s1. Data for mRNA levels of the genes of interest were normalized to that of reference control (18S ribosomal RNA, Hs99999901_s1) and were calculated using the comparative 2-ΔCT method.

### 4.7. Immunohistochemical Staining

The adipose tissue was sectioned (5 µm) using a rotary paraffin microtome (Leica, RM2255, Wetzlar, Germany). The sections were placed on IHC Microscope Slides (K8020, Dako, Santa Clara, CA, USA) and stored until their use. The sample was dewaxed with xylene and rehydrated with 100, 96, 80, 70% (5 min each) ethanol. After this step, the sample was washed in tris-buffered saline (TBS) (150 mM NaCl, 50 mM Tris-HCl, pH 7.6). The slides were placed into citrate-antigen repairing solution (0.01 M, pH 6.0) and the unmasking of the epitopes was performed using the PT LINK system (Dako) following the instructions of the manufacturer. The sample was blocked using TBS supplemented with 10% goat serum in a wet chamber for 1 h at room temperature. FD was detected using a polyclonal rabbit anti-human FD (PA5-79034, from Invitrogen) with a dilution of 1 µg/mL (overnight at 4 °C in a wet chamber). The color reaction was developed using the Mouse and Rabbit Specific HRP/DAB (ABC) Detection IHC kit (ab64264, from Abcam, Cambridge, UK). Staining was performed using a rabbit IgG (ab172730; Abcam) as an isotype control. Finally, the tissue was counterstained with hematoxylin and mounted with DPX mounting medium.

### 4.8. Statistical Analysis

Statistical analysis was performed using GraphPad Prism version 6.01 (San Diego, CA, USA). Concentrations of complement FD are represented as median with interquartile range (IQR). Mann–Whitney U and Kruskall–Wallis non-parametric tests were used for statistical analysis and Spearman Rank correlation coefficient (r) was used for correlation between variables. Receiver operating characteristic (ROC) curve analysis was used to analyze the diagnostic value of plasma FD levels as a potential biomarker. Additionally, other statistical parameters, such as sensitivity, specificity, cut-off value, positive predictive value, negative predictive value, and area under the ROC curve (AUC) with 95% confidence interval (CI), were also evaluated. *p <* 0.05 was considered statistically significant.

## Figures and Tables

**Table 1:** Clinical and demographical data of all cohorts.

	Female Sex (%)	Mean Age (Range)	C3 (mg/dL) *	C3 Low (%)	C3NeF Positive (%)	Glomerulopathy (%)	Location of Lipodystrophy	Lipodystrophy Related Mutations ^¥^
**Controls (*n* = 50)**	80%	29.88 (6–66)	112.65	None	None	NA	NA	NA
**BSS (*n* = 13)**	85%	33.69 (11–76)	63.75	69.23	69.23	23.07	Upper body	None
**C3G (*n* = 22)**	77%	26.2 (7–76)	47.8	72.72	68.18	100	None	NA
**AGL (*n* = 20)**	80%	27 (6–68)	104.56	None	None	None	Whole body	None

Abbreviations: BSS, Barraquer–Simons syndrome; C3G, C3 glomerulopathy; AGL, acquired generalized lipodystrophy; *n*, number of individuals; C3NeF, C3 nephritic factor; NA, not applicable. * Normal range for C3 levels were 75–135 mg/dL. ^¥^ Findings from the genetic screening of pathogenic variants associated to generalized (*AGPAT2, BSCL2, CAV1, PTRF, PPARG)* and partial forms (*LMNA, PPARG, PLIN1, CIDEC, LIPE, CAV1, AKT2)*.

**Table 2:** mRNA expression levels of genes of the complement system and adipogenesis pathway in subcutaneous adipose tissue samples from a patient with Barraquer–Simons syndrome and healthy controls.

	*C3*	*C5*	*CFD*	*PPARG*	*CEBPA*
Female Barraquer–Simons syndrome (arm, *n* = 1)	56.9	0.95	99.3	10.1	10.9
Female control (arm, *n* = 1)	84.8	0.67	194.0	36.5	37.7
Male control (arm, *n* = 1)	116.0	0.40	100.3	51.0	58.2

Values of mRNA expression in adipose tissue from healthy controls are expressed as the ratio of relative abundance of the mRNA of the gene of interest relative to 18S rRNA (×10^−6^). Abbreviations: *n*, number of individuals; *CFD*, Complement Factor D; *PPARG*, Peroxisome proliferator-activated receptor-γ; *CEBPA*, CCAAT/enhancer-binding protein-α.

## Data Availability

Data sharing not applicable.

## Abbreviations

AGL, acquired generalized lipodystrophy; AP, alternative pathway; AUC, Area Under the Curve; BSS, Barraquer–Simons syndrome; CGL, congenital generalized lipodystrophy; CEBPA, CCAAT/enhancer-binding protein-α; C3G, C3 glomerulopathy; C3NeF, C3 nephritic factor; CFD, Complement Factor D (gene); FD, factor D (protein); FPL, familial partial lipodystrophy; PPARG, Peroxisome proliferator-activated receptor-γ; ROC, Receiver operating characteristic.
